# Supplementary material for: Black phosphorene as a hole extraction layer boosting solar water splitting of oxygen evolution catalysts
Source: Nat Commun. 2019 May 1;10:2001. doi: 10.1038/s41467-019-10034-1 (PMC6494903; doi:10.1038/s41467-019-10034-1)
Supplement: Supplementary file 1 — Supporting information [file 41467_2019_10034_MOESM1_ESM.pdf]

Black Phosphorene as A Hole Extraction Layer Boosting Solar Water Splitting of Oxygen Evolution Catalysts

Zhang et al

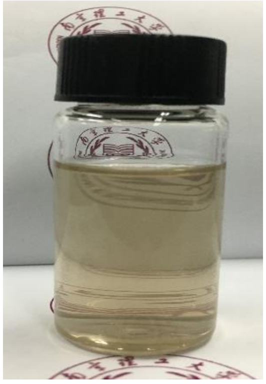

Supplementary Figure 1. BP nanosheets dispersed in isopropanol.

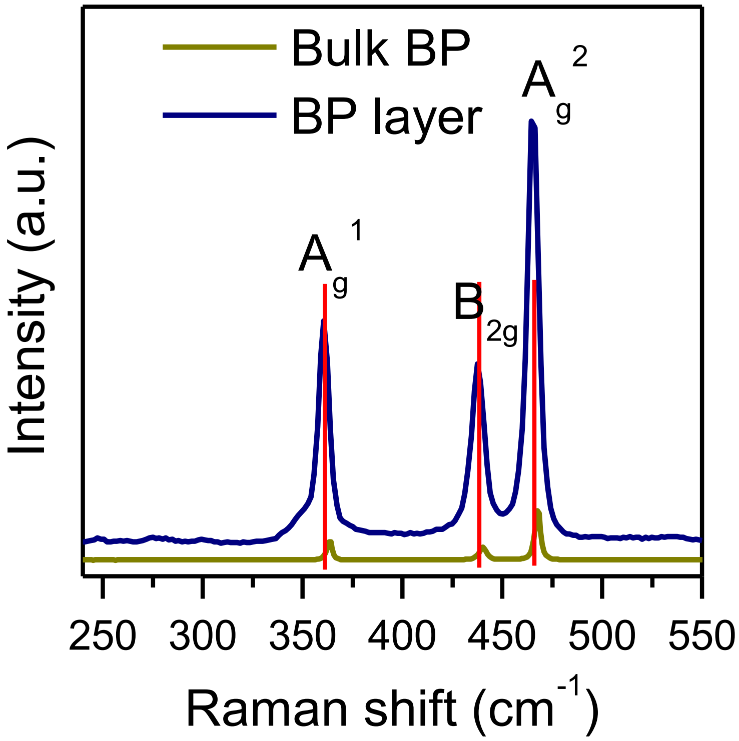

Supplementary Figure 2. Raman shifts of bulk BP and exfoliated BP layer.

Raman spectra of bulk BP and exfoliated BP sheets show three typical peaks between 300 and 500 cm^{-1}, which include an out-of-plane vibrational mode of A_g^1, two in-plane modes of B_{2g} and A_g^2 [1]. Compared to bulk BP, the Raman signals of exfoliated BP are shifted to a high number, which is in agreement with previous reports [2].

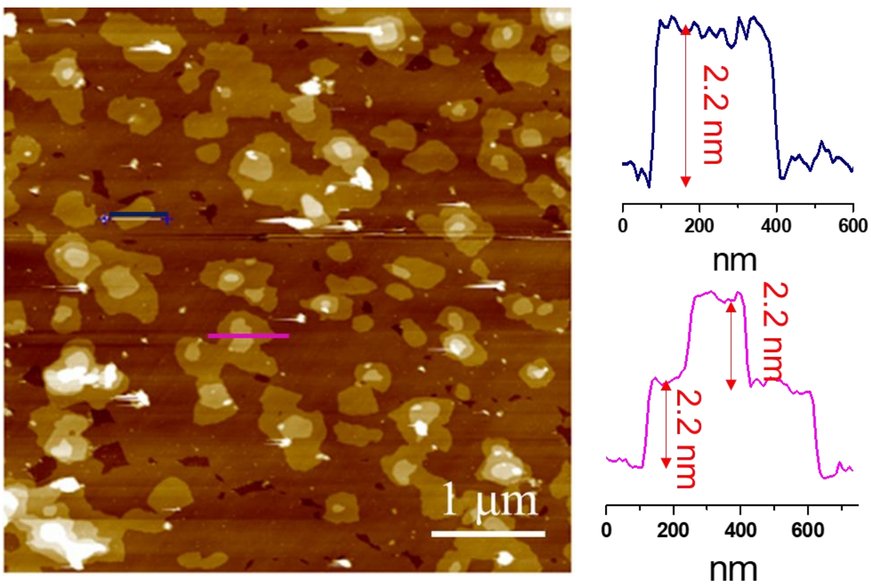

Supplementary Figure 3. AFM image of exfoliated BP layer.

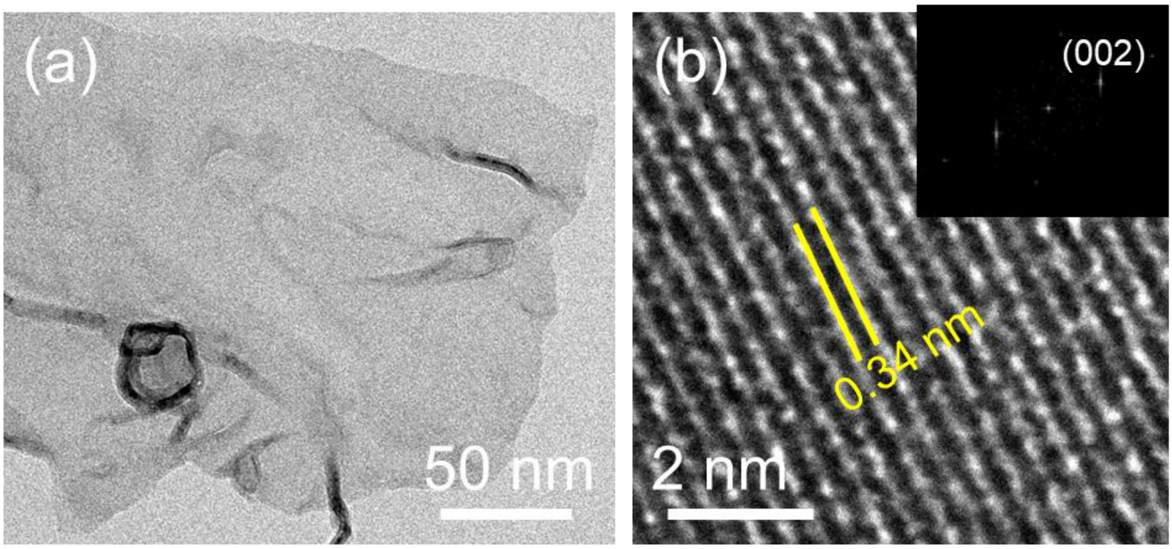

Supplementary Figure 4. TEM and HR-TEM images of exfoliated BP layer.

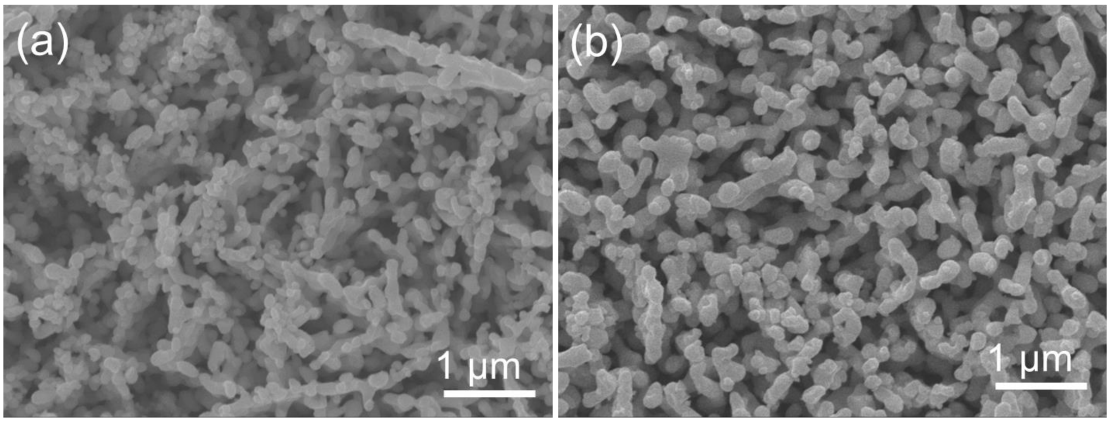

Supplementary Figure 5. SEM images of pure $BiVO_4$ and BP/$BiVO_4$ photoanodes.

The top-view SEM images indicate that the pure $BiVO_4$ photoanode has larger micropores that BP/$BiVO_4$. The changes in the porous electrode can be considered as the collapse of the structure to some extent that arises from high centripetal force through centrifuged deposition.

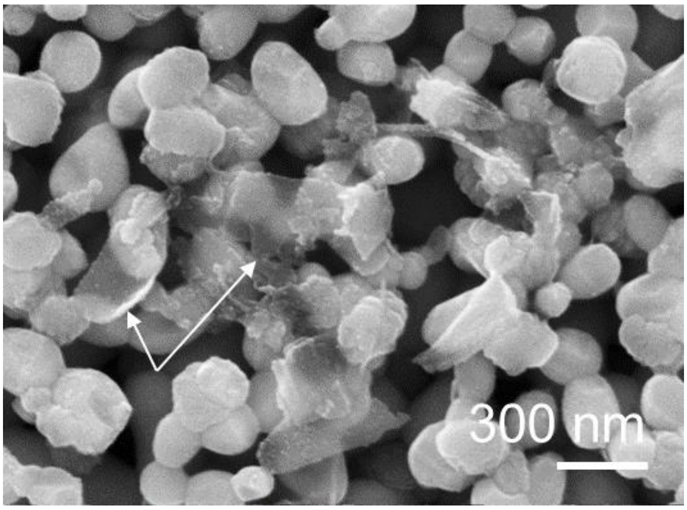

Supplementary Figure 6. SEM image of the depositing BP on $BiVO_4$ electrode by immersed $BiVO_4$ in BP dispersion.

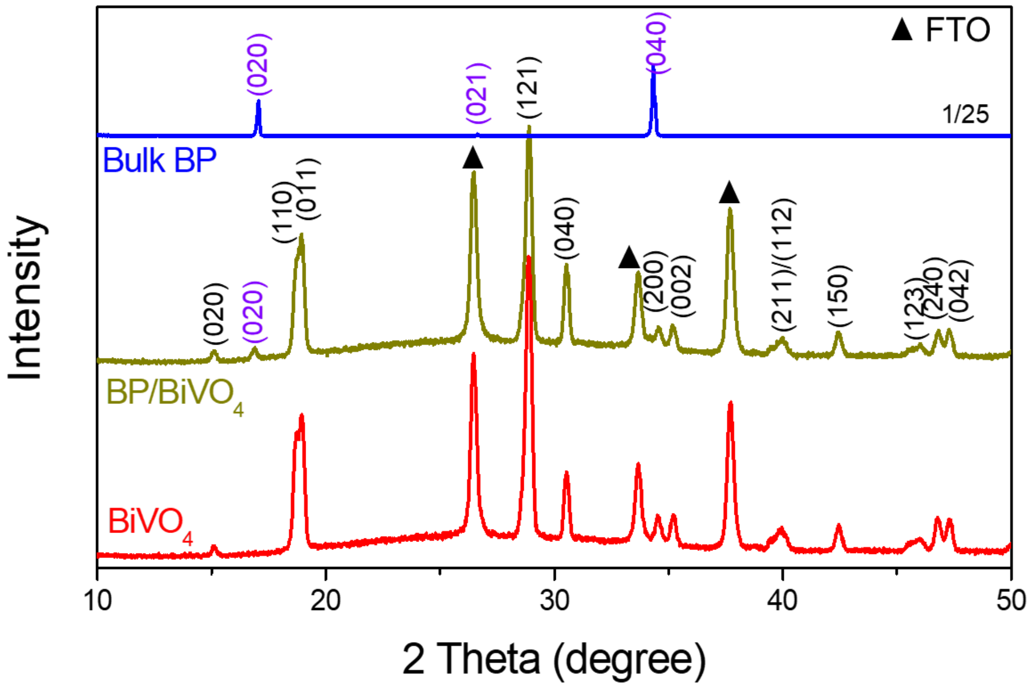

Supplementary Figure 7. XRD patterns of $BiVO_4$, BP/$BiVO_4$, and Bulk BP.

XRD pattern of BP/$BiVO_4$ clearly shows an additional peak corresponding to (002) plane of BP, as compared to that of pure $BiVO_4$.

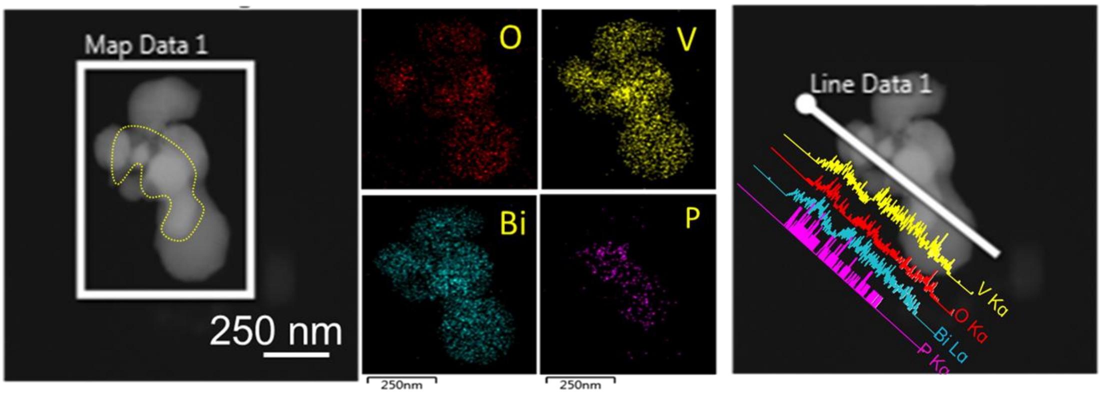

Supplementary Figure 8. HAADF-STEM-EDX image of $BP/BiVO_4$.

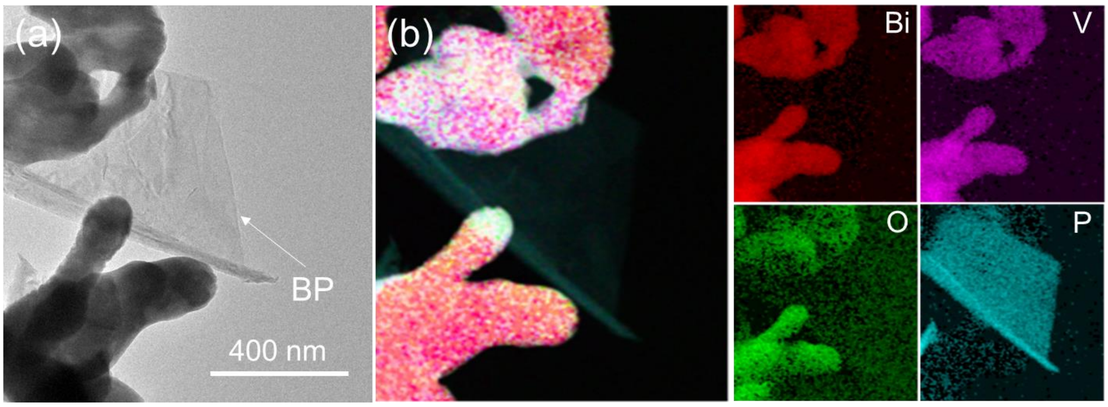

Supplementary Figure 9. TEM and HAADF-STEM-EDX element mapping images.

Figure S9 shows that the TEM and HAADF-STEM-EDX element mapping images of the depositing BP on $BiVO_4$ electrode by immersed $BiVO_4$ in BP dispersion.

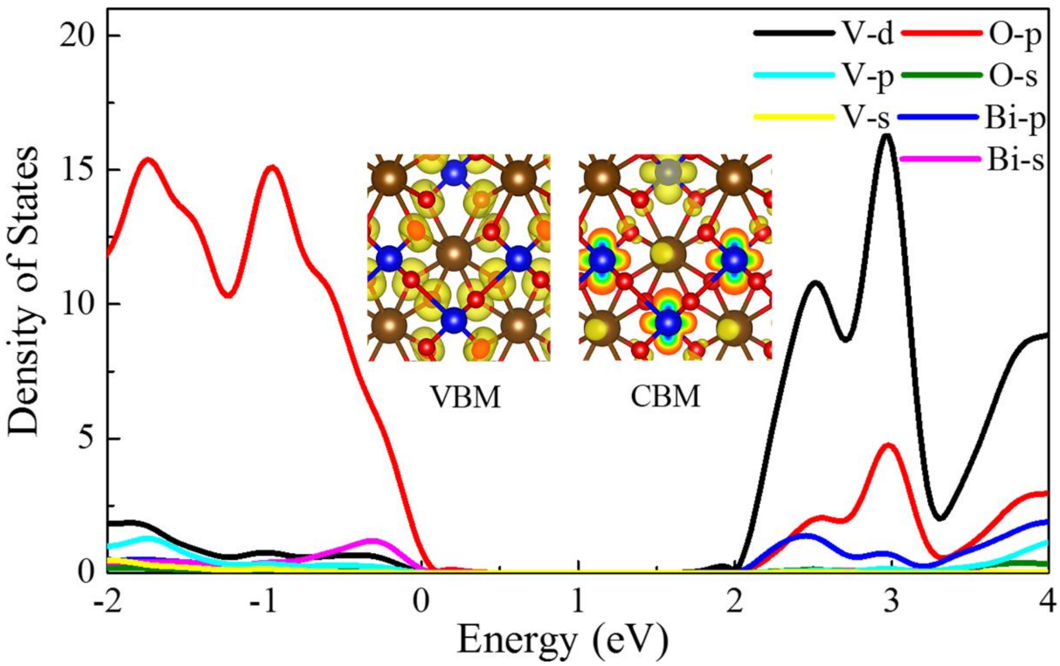

Supplementary Figure 10. Partial density of states and the corresponding charge density of valence band maximum and conduction band minimum of bulk BiVO4.

The theoretical results demonstrate that the valence band is mainly contributed by O-p orbital, together with a small contribution from Bi-s orbital mixing. The conduction band is primarily of V-d, O-p, and Bi-p with contributions from O-s orbital.

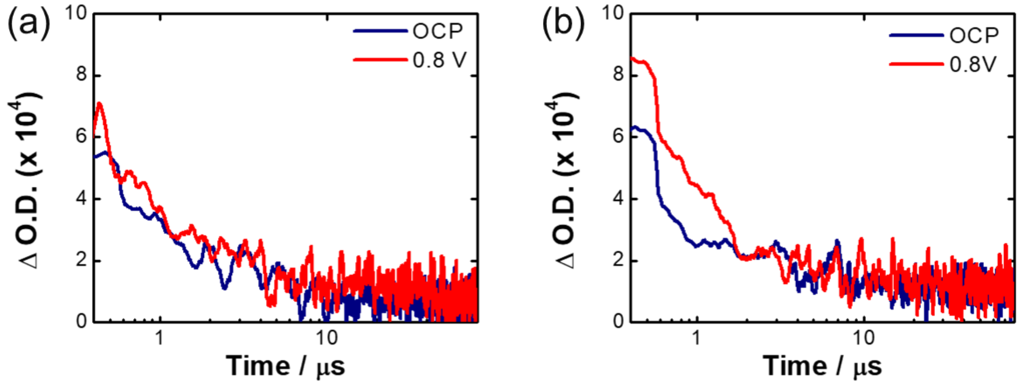

Supplementary Figure 11. In-situ transient absorption signals.

Figure 11a and 11b shows the in-situ transient absorption signals probed at 500 nm of $BiVO_4$ and BP/$BiVO_4$ photoanodes under OCP and 0.8 V vs Ag/AgCl bias, respectively.

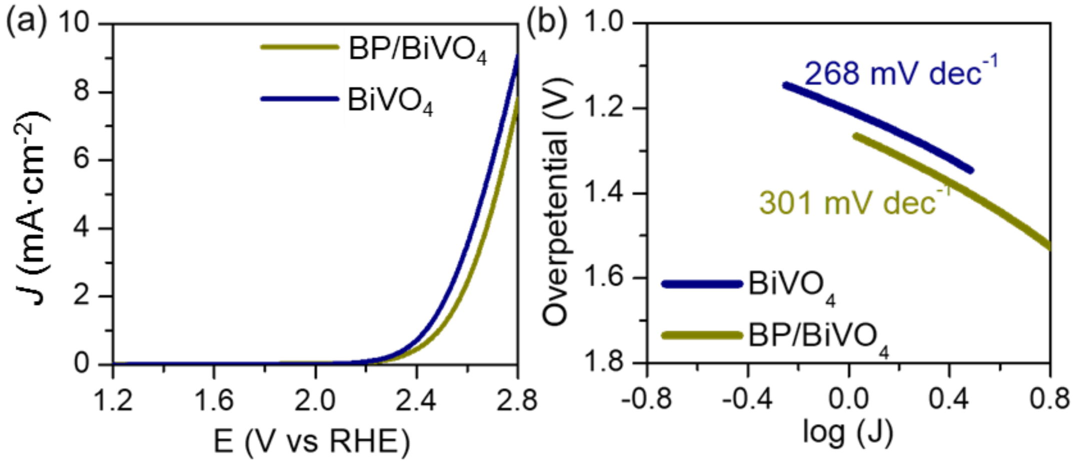

Supplementary Figure 12. The dark current density of $BiVO_4$ and $BP/BiVO_4$ with a scanning rate of 2 mV/s.

Figure S12a indicates that the OER activity of BiVO4 is lowered after deposition of BP. In Figure S12b, the estimated Tafel slope for $BiVO_4$ photoanode (268 mV dec^{-1}) is smaller than that for the $BP/BiVO_4$ photoanode (301 mV dec^{-1}), implying that the BP somewhat hinders the reaction kinetics of $BiVO_4$.

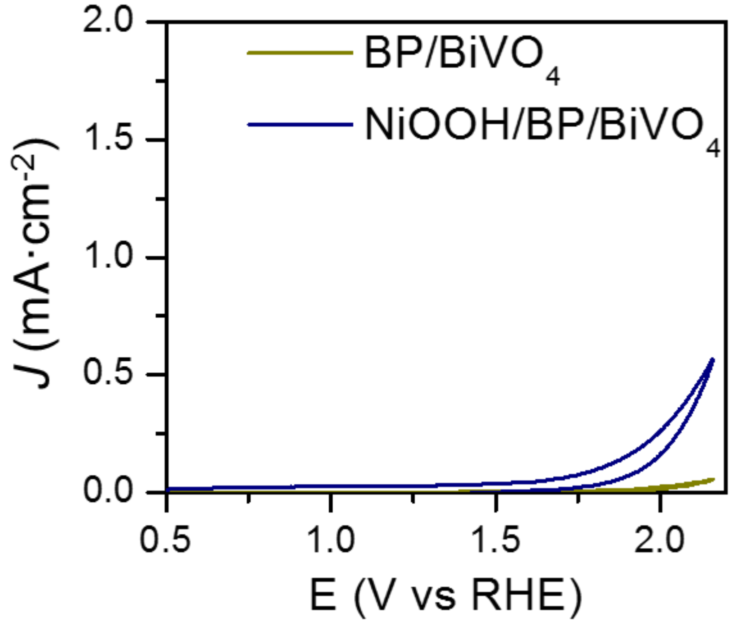

Supplementary Figure 13. CV curves of $BP/BiVO_4$ and $NiOOH/BP/BiVO_4$ with a scanning rate of 2 mV/s under dark condition.

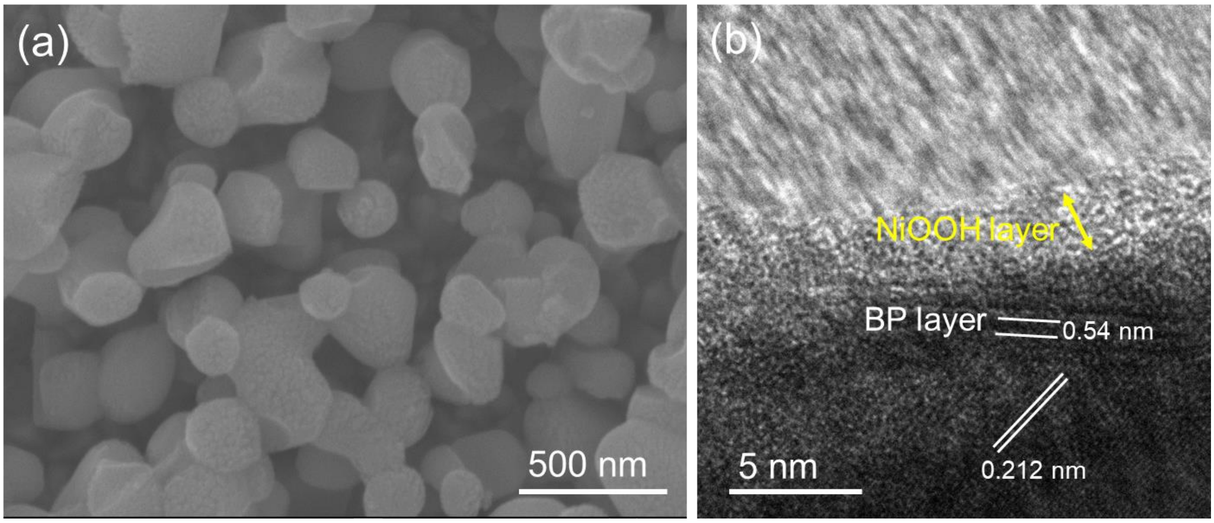

Supplementary Figure 14. SEM (a) and TEM (b) images of NiOOH/BP/$BiVO_4$ photoanode.

From the TEM image, it can be found that a 2 nm NiOOH layer is deposited on BP/$BiVO_4$ surface.

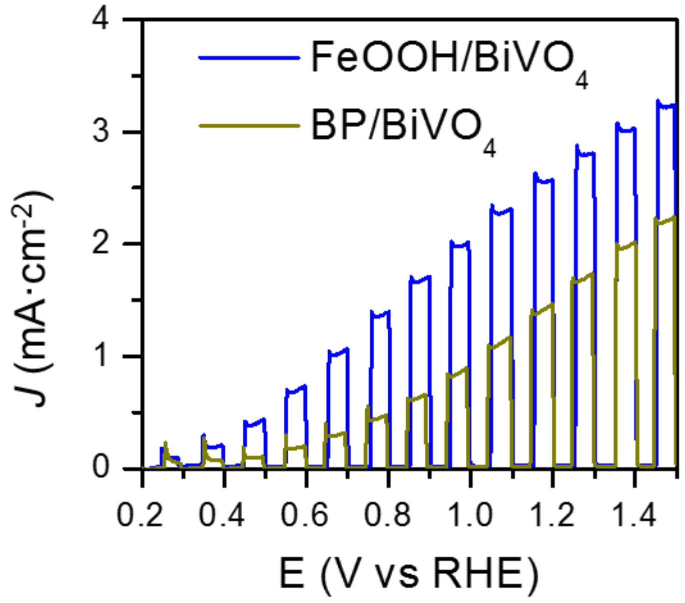

Supplementary Figure 15. Chopped *J-V* curves of $FeOOH/BiVO_4$ and $BP/BiVO_4$ in KPi electrolyte under AM 1.5 illumination.

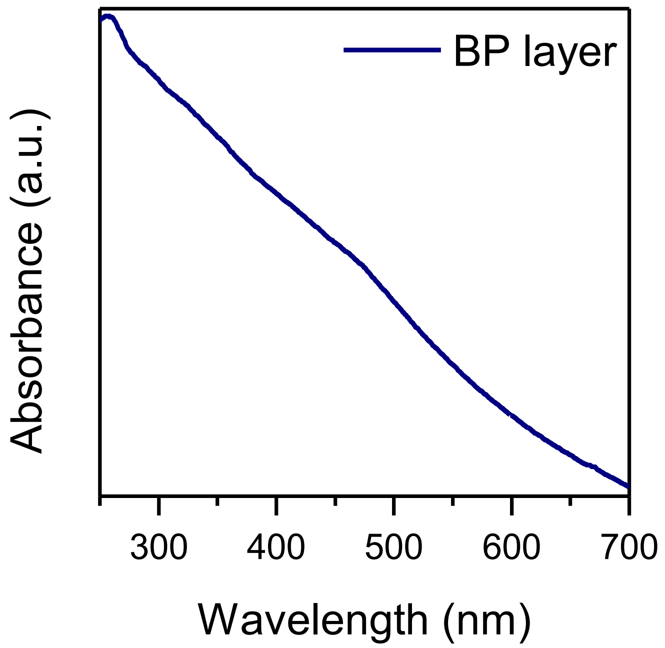

Supplementary Figure 16. UV-vis absorption spectrum of exfoliated BP layer.

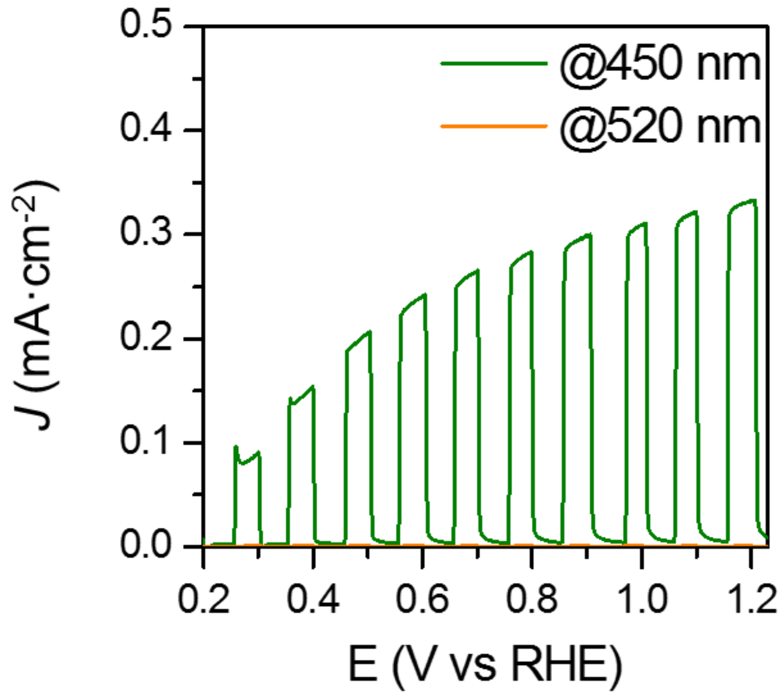

Supplementary Figure 17. Photocurrent response of BP/$BiVO_4$ photoanode in KPi electrolyte with hole scavenger (0.5M Na_2SO_3) under AM 1.5 with 450 nm and 520 nm band-pass filter.

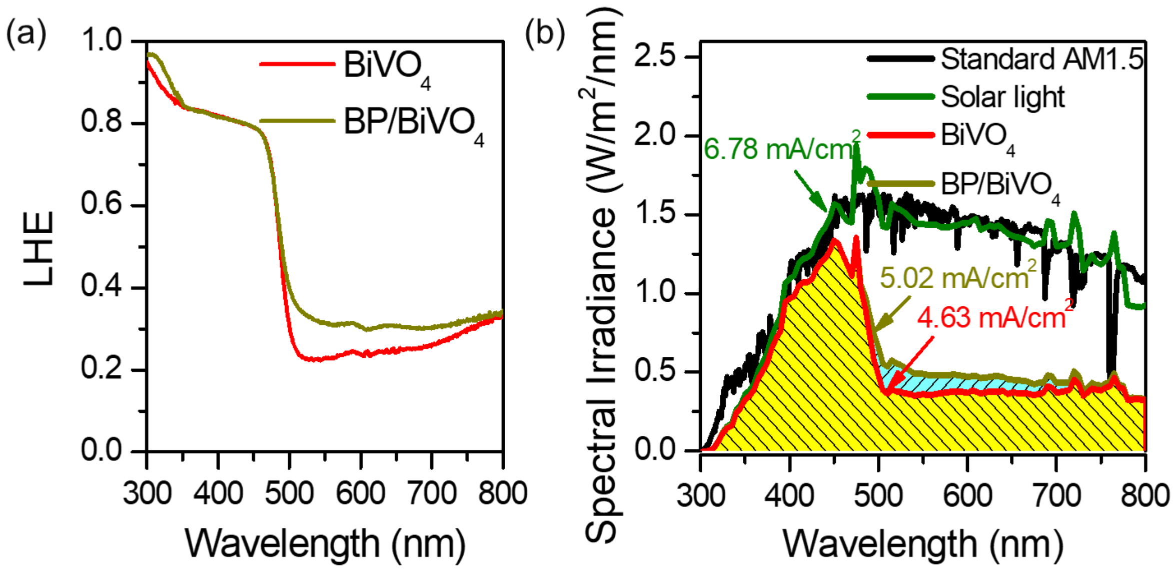

Supplementary Figure 18. (a) LHE of $BiVO_4$ and BP/$BiVO_4$ photoanodes, (b) Spectra of the solar irradiance of AM 1.5G and corresponding J_{abs} that was calculated by the LHE spectra of the $BiVO_4$ and BP/$BiVO_4$ photoanodes (300~515 nm).

Light harvesting efficiency (LHE) can be calculated by the following equation:

$$LHE = 1 - 10^{-A(\lambda)}$$

where $A(\lambda)$ is absorbance, λ is wavelength.

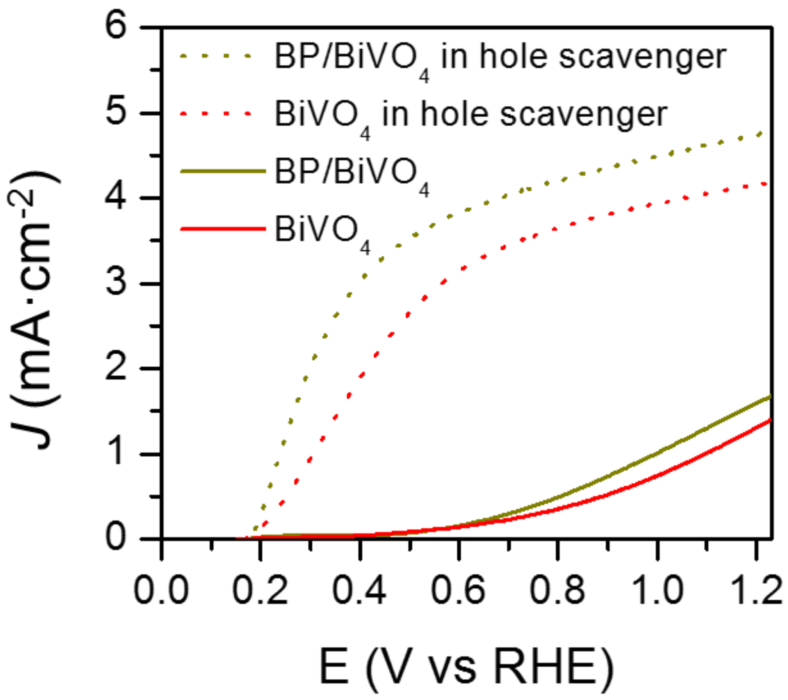

Supplementary Figure 19. J-V curves (J_{HS}) of $BiVO_4$ and BP/$BiVO_4$ measured in KPi electrolyte with and without hole scavenger (0.5M Na_2SO_3).

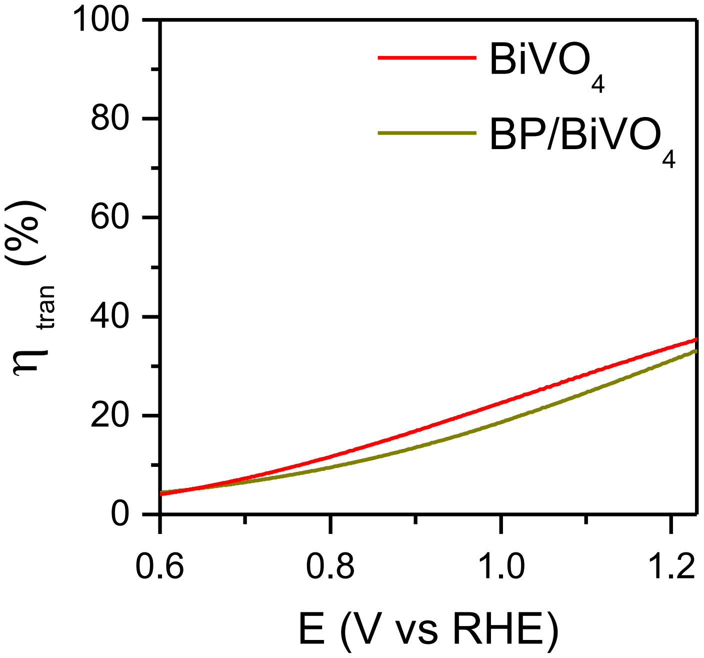

Supplementary Figure 20. Charge transfer efficiencies (η_{tran}) of $BiVO_4$ and BP/$BiVO_4$ photoanodes.

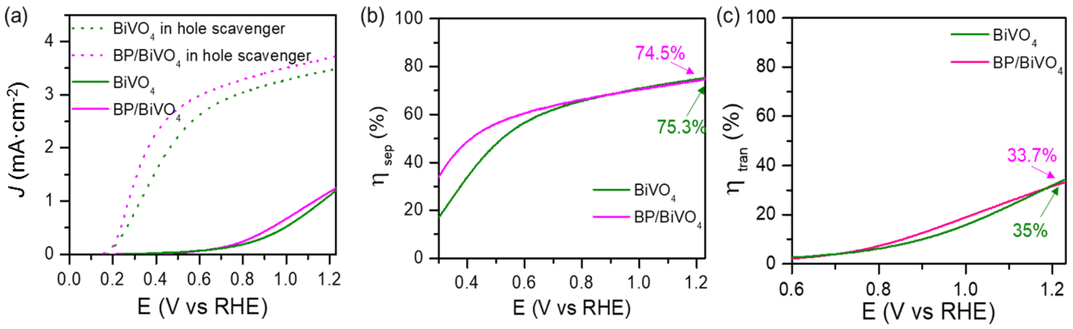

Supplementary Figure 21. (a) J-V curves of $BiVO_4$ and BP/$BiVO_4$ measured in KPi electrolyte with and without hole scavenger (0.5 M Na_2SO_3) under rear illumination. The corresponding charge separation efficiencies (b) and charger transfer efficiencies (c).

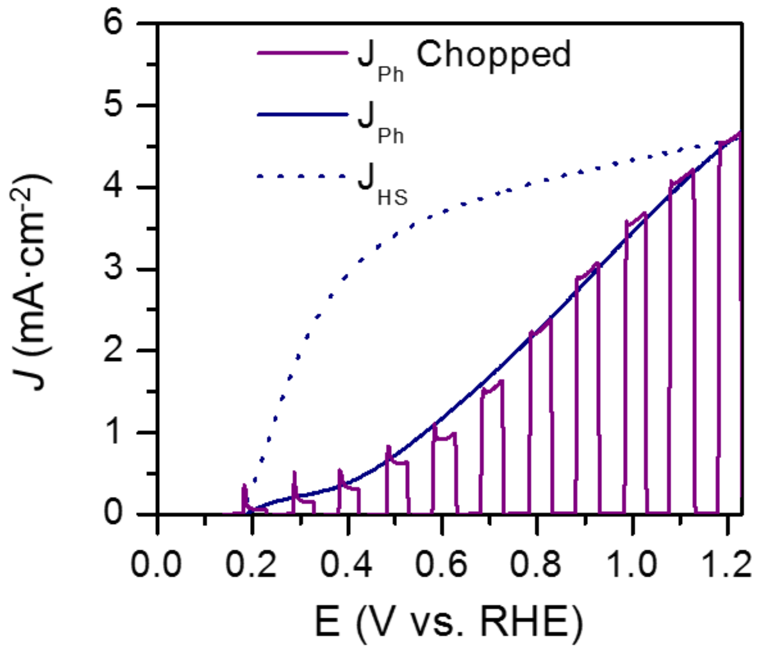

Supplementary Figure 22. J-V curves of the NiOOH/BP/$BiVO_4$ photoanodes measured in KPi electrolyte with and without hole scavenger (0.5 M Na_2SO_3).

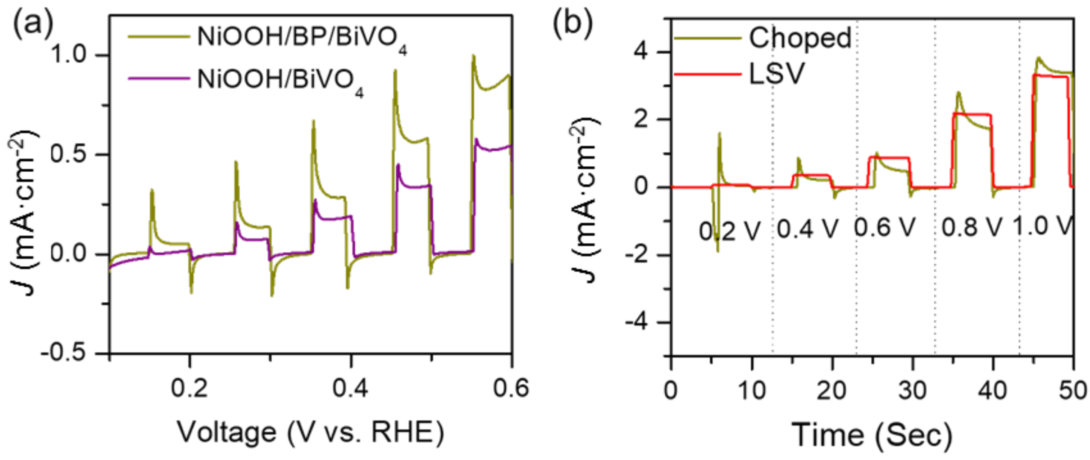

Supplementary Figure 23. (a) Chopped LSV measurements of $NiOOH/BiVO_4$ and $NiOOH/BP/BiVO_4$ photoanodes in potential ranging from 0.1 to 0.6 vs. RHE. (b) Comparison of transient-state photocurrent densities obtained by chronoamperometry (stable bias) and LSV (gradually increasing bias) in potential ranging from 0.2 to 1.0 V vs. RHE.

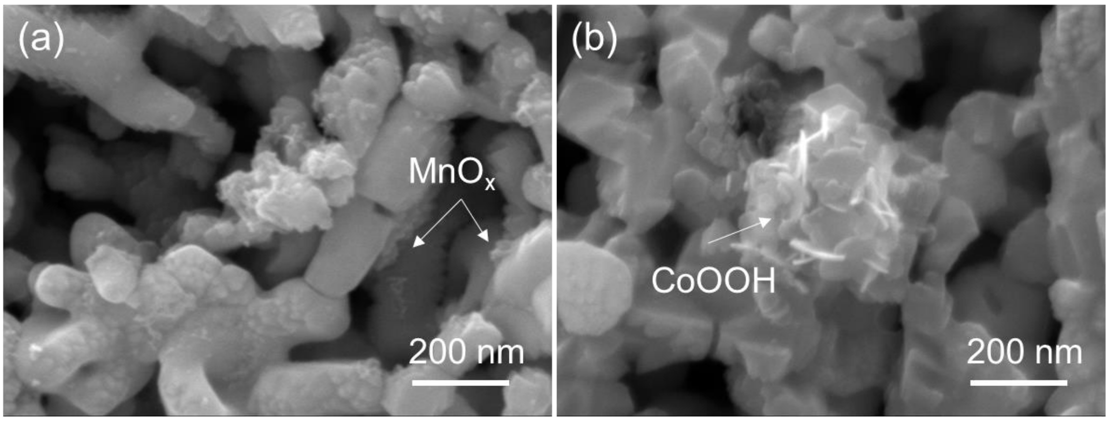

Supplementary Figure 24. SEM images of MnOx/BP/$BiVO_4$ (a) and CoOOH/BP/$BiVO_4$ (b) photoanodes.

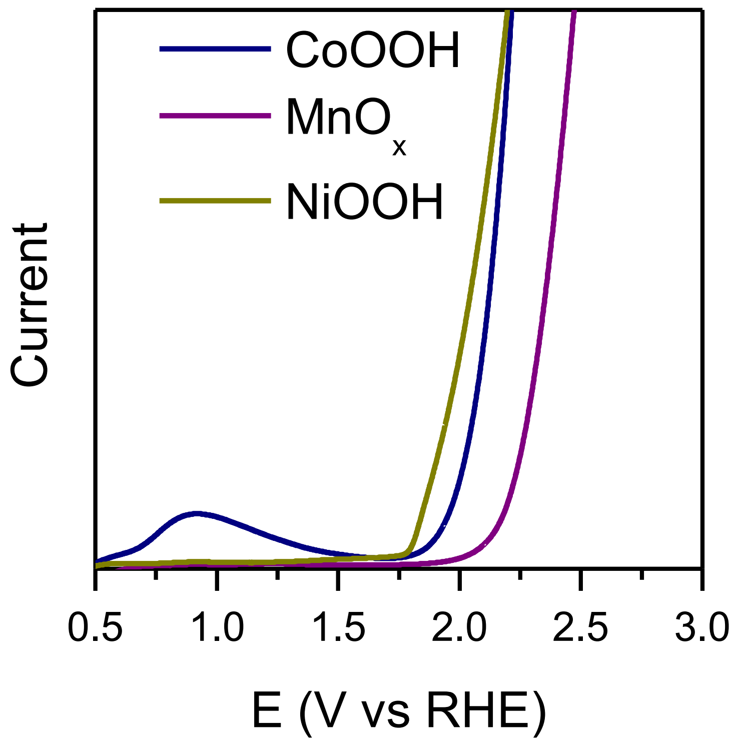

Supplementary Figure 25. OER activity of CoOOH, MnO_x, and NiOOH measured in KPi electrolyte (pH=7.1) at a scanning rate of 5 mV/s.

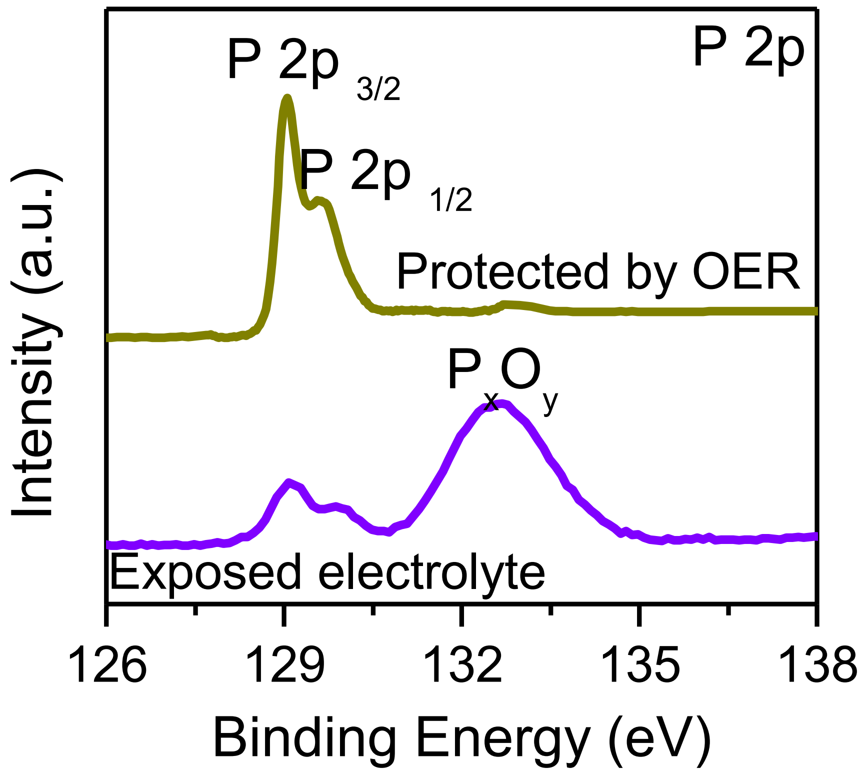

Supplementary Figure 26. P2p XPS spectrum of $BP/BiVO_4$ and $NiOOH/BP/BiVO_4$ photoanodes after long time testing. A raised P_xO_y peak can be clearly observed when the BP was directly exposed in the electrolyte, indicating considerable oxidation degree of BP during photoelectrochemical water splitting.

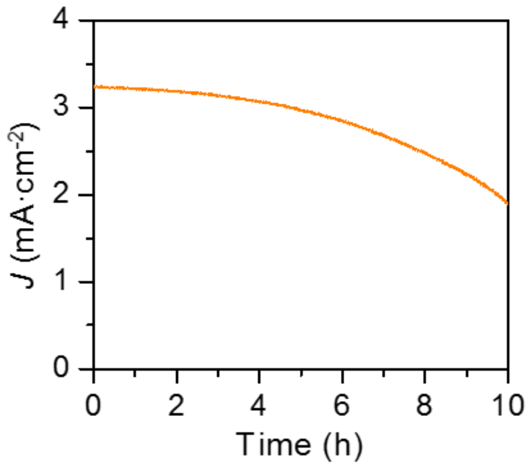

Supplementary Figure 27. Photocurrent density stability of CoOOH/BP/$BiVO_4$ photoanode measured at 1.23 V vs RHE in KPi electrolyte (pH=7.1) under AM 1.5 illumination.

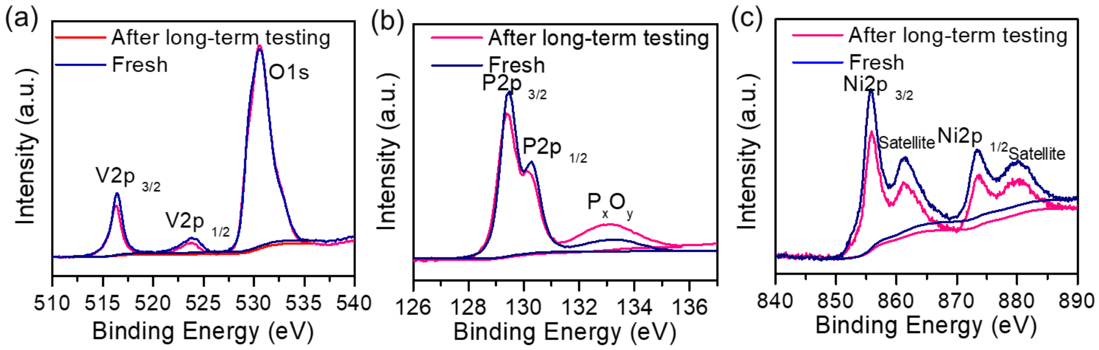

Supplementary Figure 28. Comparisons of XPS results of $NiOOH/BP/BiVO_4$ before and after long-term testing. (a) V 2p and O1s, (b) P2p and (c) Ni2p.

In Figure S28a, the O1s peak shows on detectable changes in intensity and position, whereas a slight shift of the V 2p towards lower binding energy and reduced intensity can be observed after long-term testing. The phenomenon can be tracked to the photo-charging of $BiVO_4$, by which the V in 5+ state was somehow reduced to 4+ state [3,4]. Figure S28b shows the P 2p peak before and after long-term testing. It is a fact that BP is able to be slowly oxidized during PEC testing, as evidenced by the raised P_xO_y peak. Nevertheless, the Ni 2p peaks were determined to be stable during PEC testing (Figure S28c). Overall, the $BP/BiVO_4$ protected by NiOOH layer can be relatively stable for at least 60 hours.

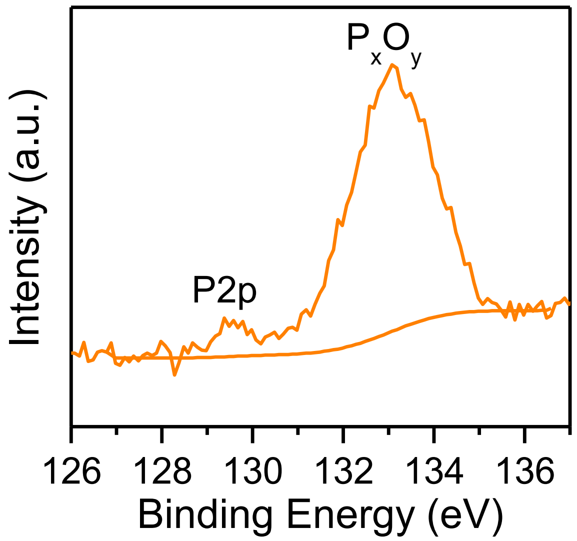

Supplementary Figure 29. P2p XPS spectrum of $CoOOH/BP/BiVO_4$ before and after long-term testing.

Supplementary Table 1. Fitting results of Nyquist plots.

Samples	R1 (Ω)	R2 (Ω)	R3 (Ω)	C_{bulk} (μF cm^{-2})	C_{trap}(μF cm^{-2})
$BiVO_4$	11.6	3200		35.2	29.7
BP/$BiVO_4$	12.4	1084		32.9	328.9
NiOOH/FeOOH/$BiVO_4$	10.3	204	1412	30.2	132
NiOOH/BP/$BiVO_4$	10.2	162	1416	32.2	567.5

Supplementary Table 2. Photocurrent density at 1.23 V vs RHE and enhancement factors.

Samples	J (mA/cm^2)	Enhancement factors (with BP/without BP)
$NiOOH/BiVO_4$	3.03	1.48
$NiOOH/BP/BiVO_4$	4.48	
$CoOOH/BiVO_4$	2.23	1.51
$CoOOH/BP/BiVO_4$	3.37	
$MnO_x/BiVO_4$	1.85	1.74
$MnO_x/BP/BiVO_4$	3.22	

Supplementary Table 3. Recent progress on PEC performance of various OEC/$BiVO_4$ photoanodes.

Category	Photoanode	OEC	Photocurrent density (mA/cm^2 @ 1.23 V vs RHE)	Ref
Pristine OEC/$BiVO_4$	$BiVO_4$	Co-pi	3.6	S4
	$BiVO_4$	FeOOH	~2.0	S5
	$BiVO_4$	CoOOH	4.0	S6
	$BiVO_4$	Ni-Bi	1.8	S7
	$BiVO_4$	Co_3O_4	2.71	S8
	$BiVO_4$	Co-Bi	3.2	S9
	$BiVO_4$	Fe-Ni LDH	1.21	S10
	$BiVO_4$	NiB	3.47	S11
	$BiVO_4$	Co−La LDH	~2.0	S12
	$BiVO_4$	NiOOH/FeOOH	4.8	S13
	$BiVO_4$	CoFe(OH)x	2.48	S14
	$BiVO_4$	$NiFe_x$-Bi	~4	S15
	$BiVO_4$	Molecular Co_4O_4 Cubane	~5	S16
Modified OEC/BiVO4	N doped $BiVO_4$	NiOOH/FeOOH	~5	S17
	$BiVO_4$	p-NiO/CoOx	3.5	S18
	$BiVO_4$	Oxygen defect FeOOH	4.3	S19
	$BiVO_4$	In_2O_3 thin layer/CoOOH	3.4	S20
	$BiVO_4$	TiO_2 thin layer/Ir-COOH	~2.7	S21

	$BiVO_4$	BP/NiOOH	4.48	Our work

Supplementary References

1. Li, L. et. al. Black phosphorus field-effect transistors. *Nat. Nanotech.* **9,** 372 (2014).
2. Kang, J. et. al. Stable aqueous dispersions of optically and electronically active phosphorene. *Proc. Natl. Acad. Sci.* **113,** 11688–11693 (2016).
3. Trześniewski, B. et. al. Near-complete suppression of surface losses and total internal quantum efficiency in $BiVO_4$ photoanodes. *Energy Environ. Sci.* **10**, 1517−1529 (2017).
4. Trześniewski, B. & Smith, W. Photocharged $BiVO_4$ photoanodes for improved solar water splitting. *J. Mater. Chem. A* **4**, 2919−2926 (2016)
5. Abdi, F. F. & van de Krol, R. Nature and light dependence of bulk recombination in Co-Pi-catalyzed $BiVO_4$ photoanodes. *J. Phys. Chem. C* **116,** 9398–9404 (2012).
6. Seabold, J. A. & Choi, K.S. Efficient and stable photo-oxidation of water by a bismuth vanadate photoanode coupled with an iron oxyhydroxide oxygen evolution catalyst. *J. Am. Chem. Soc.* **134,** 2186–2192 (2012).
7. Tang, F. Cheng, W. Su, H. Zhao, X. & Liu, Q. Smoothing surface trapping states in 3D coral-like CoOOH-wrapped-$BiVO_4$ for efficient photoelectrochemical water oxidation. *ACS Appl. Mater. Interfaces* **10,** 6228–6234 (2018).
8. Choi, S. K. Choi, W. & Park, H. Solar water oxidation using nickel-borate coupled $BiVO_4$ photoelectrodes. *Phys. Chem. Chem. Phys.* **15,** 6499–6507 (2013).
9. Chang, X. Wang, T. Zhang, P. Zhang, J. Li, A. & Gong, J. Enhanced surface reaction kinetics and charge separation of p-n heterojunction $Co_3O_4/BiVO_4$

photoanodes. *J. Am. Chem. Soc.* **137,** 8356–8359 (2015).

10. Wang, S. Chen, P. Yun, J. H. Hu, Y. & Wang, L. An electrochemically treated $BiVO_4$ photoanode for efficient photoelectrochemical water splitting. *Angew.Chem. Int. Ed.* **56,** 8500–8504 (2017).
11. Zhu, Y. et. al. Interface engineering of 3D $BiVO_4$/Fe-based layered double hydroxide core/shell nanostructures for boosting photoelectrochemical water oxidation. *J. Mater. Chem. A* **5,** 9952–9959 (2017).
12. Dang, K. Chang, X. Wang, T. & Gong, J. Enhancement of photoelectrochemical oxidation by an amorphous nickel boride catalyst on porous $BiVO_4$. *Nanoscale* **9,** 16133–16137 (2017).
13. Chhetri, M. Dey, S. & Rao, C. N. R. Photoelectrochemical oxygen evolution reaction activity of amorphous Co-La double hydroxide-$BiVO_4$ fabricated by pulse plating electrodeposition. *ACS Energy Lett.* **2,** 1062–1069 (2017).
14. Kim, T. W. & Choi, K. S. Nanoporous $BiVO_4$ photoanodes with dual-layer oxygen evolution catalysts for solar water splitting. *Science* **343,** 990–994 (2014).
15. Liu, W. Liu, H. Dang, L. Zhang, H. Wu, X. Yang, B. Li, Z. Zhang, X. Lei, L. & Jin, S. Amorphous cobalt-iron hydroxide nanosheet electrocatalyst for efficient electrochemical and photo-electrochemical oxygen evolution. *Adv. Funct. Mater.* **27,** 1603904 (2017).
16. Kuang, Y. Jia, Q. Nishiyama, H. Yamada, T. Kudo, A. & Domen, K. A front-illuminated nanostructured transparent $BiVO_4$ photoanode for>2% efficient water splitting. *Adv. Energy Mater.* **6,** 1501645 (2016).
17. Wang, Y. et. al. Highly efficient photoelectrochemical water splitting with an

immobilized molecular Co_4O_4 cubane catalyst. *Angew. Chem. Int. Ed.* **56,** 6911–6915 (2017).

18. Kim, T. W. Ping, Y. Galli, G. A. & Choi, K. S. Simultaneous enhancements in photon absorption and charge transport of bismuth vanadate photoanodes for solar water splitting. *Nat. Commun.* **6,** 8769 (2015).

19. Zhong, M. et. al. Surface modification of CoO_x loaded $BiVO_4$ photoanodes with ultrathin p-type NiO layers for improved solar water oxidation. *J. Am. Chem. Soc.* **137,** 5053–5060 (2015).

20. Zhang, B. Wang, L. Zhang, Y. Ding, Y. & Bi, Y. Ultrathin FeOOH nanolayers with abundant oxygen vacancies on $BiVO_4$ photoanodes for efficient water oxidation. *Angew. Chem. Int. Ed.* **57,** 2248–2252 (2018).

21. Qiu, W. Huang, Y. Tang, S. Ji, H. & Tong, Y. thin-layer indium oxide and cobalt oxyhydroxide cobalt-modified $BiVO_4$ photoanode for solar-assisted water electrolysis. *J. Phys. Chem. C.* **121,** 17150–17159 (2017).

22. Kan, M. et. al. A highly efficient nanoporous $BiVO_4$ photoelectrode with enhanced interface charge transfer Co-catalyzed by molecular catalyst. *Applied Catalyst. B: Environ.* **225,** 504–511 (2018).
